# Identification of novel Jingmen tick virus from parasitic ticks fed on a giant panda and goats in Sichuan Province, southwestern China

**DOI:** 10.3389/fmicb.2023.1179173

**Published:** 2023-06-14

**Authors:** Lin Huang, Shunshuai Liu, Lu Chen, Fei Wang, Ping Ye, Luoyuan Xia, Baogui Jiang, Hao Tang, Qingyu Zhang, Xiangdong Ruan, Weijun Chen, Jiafu Jiang

**Affiliations:** ^1^Department of Epidemiology and Biostatistics, School of Public Health, Anhui Medical University, Hefei, China; ^2^State Key Laboratory of Pathogen and Biosecurity, Beijing Institute of Microbiology and Epidemiology, Beijing, China; ^3^College of Life Sciences, Fujian Agriculture and Forestry University, Fuzhou, China; ^4^Beijing Macro & Micro-test Bio-Tech Co., Ltd., Beijing, China; ^5^Sichuan Forestry and Grassland Pest Control and Quarantine Station, Chengdu, China; ^6^Wolong National Natural Reserve Administration Bureau, Wenchuan, China; ^7^Academy of Inventory and Planning, National Forestry and Grassland Administration, Beijing, China; ^8^BGI PathoGenesis Pharmaceutical Technology, BGI-Shenzhen, Shenzhen, China; ^9^College of Life Sciences, University of Chinese Academy of Sciences, Beijing, China

**Keywords:** Jingmen virus group, tick borne disease, Sichuan tick virus, Sichuan Province, giant panda

## Abstract

**Introduction:**

Tick-borne viruses (TBVs) pose a significant risk to the health of humans and other vertebrates. A class of multisegmented flavi-like viruses, Jingmen tick virus (JMTV) was first discovered in Rhipicephalus microplus ticks collected from Jingmen of Hubei Province, China in 2010. JMTV has been confirmed to have a relatively wide distribution in vectors and hosts and is associated with human diseases.

**Methods:**

Parasitic and host-seeking ticks were collected in Wolong Nature Reserve, Sichuan Province. Total RNA was extracted and then enriched the viral RNA. The DNA library was constructed and then were sequenced with MGI High-throughput Sequencing Set (PE150). After the adaptor sequences,low-quality bases and host genome were removed, resulting reads classified as a virus were subsequently de novo assembled into contigs, which were then compared to the NT database. Those annotated under the kingdom virus were initially identified as potential virus-associated sequences. Phylogenetic and Reassortment analysis of sequences were performed using MEGA and SimPlot software, respectively.

**Results and discussion:**

Two host-seeking ticks and 17 ticks that fed on giant pandas and goats were collected. Through high-throughput sequencing, whole virus genomes were attained from four tick samples (PC-13, PC-16, PC-18, and PC-19) that shared 88.7–96.3% similarity with known JMTV. Phylogenetic tree showed that it was a novel JMTV-like virus, referred to as Sichuan tick virus, which also had the signals of reassortment with other JMTV strains, suggesting a cross-species transmission and co-infection of segmented flavi-like viruses among multiple tick hosts.

**Conclusion:**

We discovered and confirmed one new Jingmen tick virus, Sichuan tick virus. Further investigation is required to determine the pathogenicity of Sichuan tick virus to humans and animals, as well as its epidemiological characteristics in nature.

## 1. Introduction

In recent decades, emerging tick-borne viruses (TBVs) has been increasing and widely distributed all over the world. Since the confirmation of the first TBV, the Louping ill virus found in Scottish sheep, over 80 years ago (Jeffries et al., 2014), at least 160 such viruses have been found to be transmitted by ticks (Bartíková et al., 2017). Among the confirmed TBVs are 9 families and at least 12 genera, as well as other unassigned members (Shi et al., 2018). TBVs feature a wide range of hosts, including cattle, sheep, humans, rodents, and horses, some of which can cause human diseases and losses in relation to animal husbandry and agriculture. Thus far, at least 5 families, namely *Nairoviridae, Phenuiviridae, Flaviviridae, Orthom-yxoviridae* and *Reoviridae*, and 15 species of TBVs that cause human diseases worldwide have been found (Liu et al., 2020; Ma et al., 2021). In China, emerging TBVs have been reported to cause multiple human diseases: tick-borne encephalitis virus (TBEV) (Xing et al., 2017), severe fever with thrombocytopenia syndrome virus (SFTSV) (Yu et al., 2011), Crimean-Congo hemorrhagic fever virus (CCHFV) (Sun et al., 2011), Jingmen tick virus (JMTV) (Qin et al., 2014; Jia et al., 2019), Alongshan virus (ALSV) (Wang et al., 2019), and Songling virus (SGLV) (Ma et al., 2021). The TBVs associated with human diseases are transmitted to humans through tick bites, causing varying degrees of symptoms, while other TVBs pose unclear risks to public health.

Jingmen tick virus (JMTV) is a novel tick-borne RNA virus that was identified in a pool of *Rhipicephalus microplus* collected from the Jingmen region of Hubei Province, China in 2010 (Qin et al., 2014). The viral genome of JMTV, the segmented flavi-like virus, consists of four segments. Two segments encode non-structural proteins that are genetically related to the NS3 and NS5 sequences of the genus *Flavivirus*, while the other two segments, which encode structural proteins, are completely unique suggesting that they might have originated from a yet uncharacterized virus (Qin et al., 2014). In 2014, partial protein sequences of Mogiana tick virus (MGTV) from Brazilian *Rhipicephalus microplus* were first reported as a novel tick-borne virus of the *Flaviviridae* family before JMTV was initially identified (Maruyama et al., 2014). Notably, the sequences of MGTV shared 92.4%-96.8% amino acid identity and 88%-90.3% nucleotide similarity with JMTV, which suggests they may be the same virus (Maruyama et al., 2014; Villa et al., 2017). Subsequently, JMTV has been detected in arthropods (ticks and mosquitoes) and vertebrates (rodents, humans, monkeys, cattle, and tortoises) from China, Uganda, Brazil, Kosovo, French, Turkey, and Kenya (Webster et al., 2015; Ladner et al., 2016; Shi et al., 2016; Emmerich et al., 2018; Souza et al., 2018; Temmam et al., 2019). More significantly, JMTV has been proven to infect human and cause mild to severe diseases through high-throughput sequencing of skin biopsies and blood samples (Jia et al., 2019).

The jingmenviruses group, which was identified recently, tentatively refers to segmented flavi-like viruses that have not been formally classified by the International Committee on Taxonomy of Viruses (ICTV) in 2015 (Shi et al., 2016; Temmam et al., 2019). This group is presently divided into two phylogenetic clades. The viruses of one clade are associated with ticks and vertebrates, such as JMTV, MGTV (from ticks in Brazil), ALSV (from ticks and humans in China, Finland, and Russia), and Yanggou tick virus (from ticks in China) (Qin et al., 2014; Shi et al., 2016; Wang et al., 2019; Colmant et al., 2022). The second clade comprises insect- and other host-associated jingmenviruses, such as Guaico Culex virus (GCXV) (from mosquitoes in Panama, Peru, and Trinidad), Wuhan aphid virus (WHAV) (from aphid in China), Wuhan flea virus (WHFV) (from flea in China), and Shuangao insect virus 7 (SAIV7) (from insects in China) (Sun et al., 2011; Colmant et al., 2022). Additionally, Heilongjiang tick virus (HLJTV), Amblyomma virus, Guangxi tick virus (GXTV), and *Rhipicephalus* associated flavi-like virus also classified as jingmenviruses and share a high similarity with JMTV strain SY84, with 93.06–94.39%, 92.81–94.52%, 92.8–94.5%, and 93–99% identity respectively (Liu, 2018; Shi et al., 2021). Therefore, it can be seen that jingmenviruses have been emerging around the world with a diversity of hosts, members of which lead to severe diseases. Thus, it is crucial to conduct jingmenviruses research in the coming years to deal with emerging related diseases.

In China, JMTV has been detected in various hosts, such as arthropods, humans, bats, rodents and mammals from Hubei, Fujian, Heilongjiang, Yunnan, Guizhou, Henan, Guangxi and Zhejiang provinces (Guo et al., 2020; Colmant et al., 2022). However, there is a lack of data regarding JMTV infections in Sichuan Province, where the national-level protected animal, giant pandas, is often parasitized by many ticks. To investigate the tick virome in Sichuan Wolong National Nature Reserve, host-seeking ticks and parasitic ticks from giant pandas and goats were collected in the area. High-throughput sequencing was then conducted in the collected ticks to determine the tick virome. The objectives of the study were to understand the potential threat of JMTV-like viruses on public health to local residents, as well as protected animals and livestock.

## 2. Materials and methods

### 2.1. Sample collection

**Figure 1:**
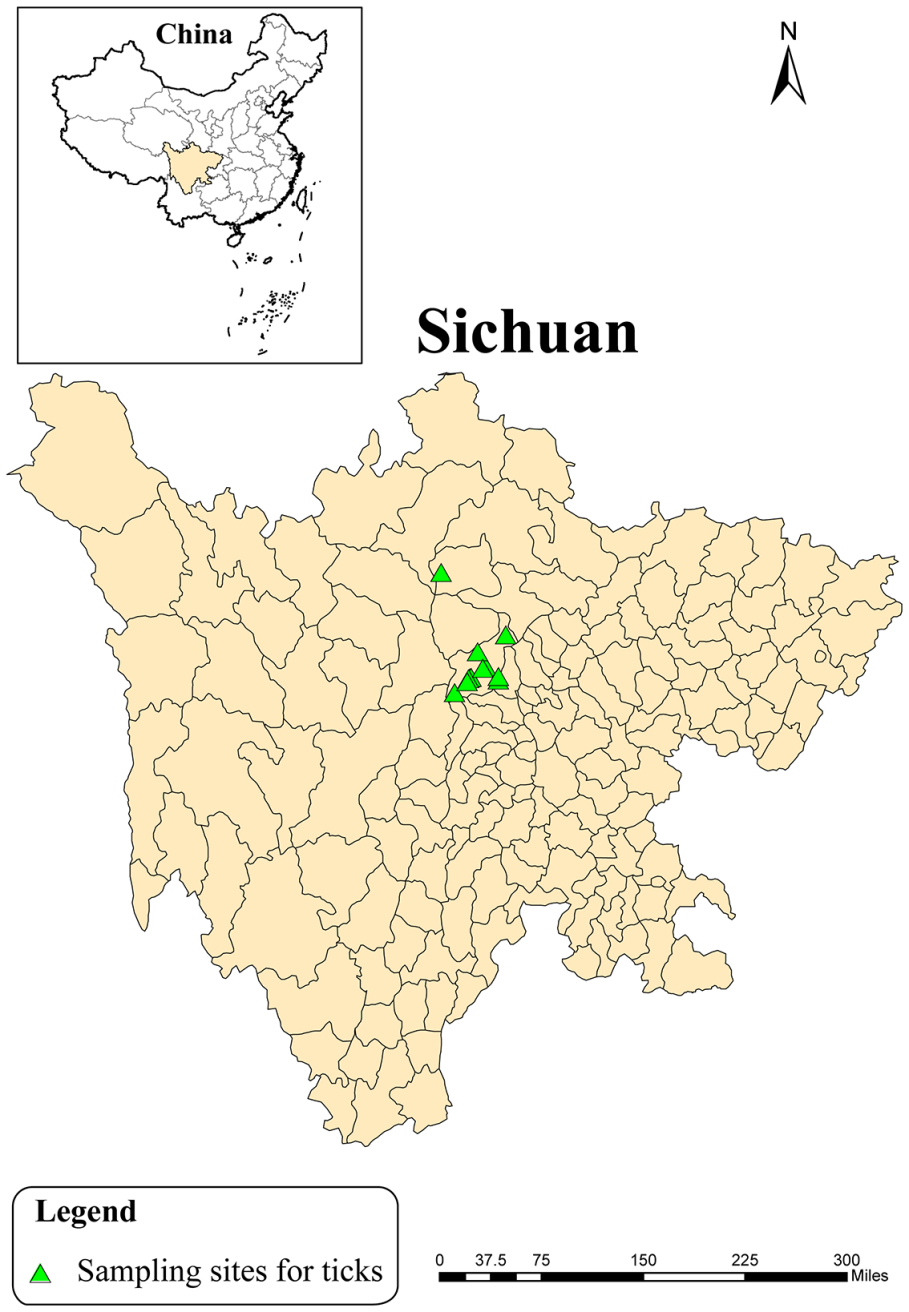
Location of sampling sites of ticks in Wolong Nature Reserve, Sichuan Province, 2020.

Parasitic and host-seeking ticks were collected in Wolong Nature Reserve, Sichuan Province using different methods in 2020 (Figure 1). The parasitic ticks were carefully removed from giant pandas and goats by using tweezers, while host-seeking ticks were collected from vegetation by flagging. The collected ticks were stored in porous centrifuge tubes with moistened sterile filter paper. Subsequently, morphological identification was performed under a stereomicroscope. The collected ticks were kept in an artificial climate chamber at 20°C until further testing was conducted.

### 2.2. RNA extraction, library preparation, and sequencing

The RNA of collected live ticks was extracted using AllPrep DNA/RNA Mini Kit (cat.no.80204, QIAGEN) according to the manufacturer’s instructions. The ticks were categorized into different groups, such as one engorged tick as a pool, 2–5 non-engorged ticks (around 20 mg) as one pool, and eggs of the engorged tick as one pool.

A Nucleic Acid Microbes Purification kit (KAPA RNA HyperPrep Kit with RiboErase, KK8561#, Roche) was also used to enrich the viral RNA from total RNA samples. The remaining RNA was fragmented to about 280 bp and then reverse-transcribed into cDNA. Next, a second strand synthesis was conducted by using a Reverse transcription kit (miScript II RT Kit, Cat.No.218161, QIAGEN). Through the synthetic double-stranded DNA, a DNA library was constructed through end-repair, UDI (unique dual-index) adaptor-ligation, and PCR amplification.

The constructed library was qualified with a Qubit® 4.0 Fluorometer (ThermoFisher, Foster City, CA, United States). The library fragment size was analyzed with Qsep-100 (Hangzhou Houze Biotechnology Co., Ltd.). The Library Quant Kit (KAPA, Roche, Rotkreuz, Switzerland) was used to accurately quantify the library for screening qualified libraries. The qualified library was pooled at 3 nmol each. After circularization and generating DNA nanoballs (DNBs), the resulting libraries were sequenced with MGI High-throughput Sequencing Set (PE150) on MGISEQ-2000 platforms (MGI, Shenzhen, China).

### 2.3. Quantification of virus abundance

Adaptor sequences and low-quality bases were removed from raw sequencing reads by the fastp program (v0.23.1) to obtain clean data. The process of sequence mapping/alignment in the clean reads uses HISAT2 (v2.1.0) based on the hierarchical indexing of host genomes with default parameters to remove the host genome. The unaligned reads were used for microbial taxonomy *via* the Kraken2 program (v2.0.9-beta) taxonomic classifier based on a custom extended RefSeq database. The outputs generated by Kraken2 were transferred to the Bracken program to acquire more accurate estimations using abundance and diversity.

### 2.4. Viral contigs assembly and annotation

The resulting reads classified as a virus by Kraken2 were subsequently *de novo* assembled into contigs using the Megahit program (v1.2.9) (Vijaykrishna et al., 2015) with default parameters. The assembled contigs were then compared to the NT database using the blastn program (v0.9.21) (Ladner et al., 2016) with an *E*-value cut-off of 1 × 10^−5^ to identify viral sequences. Taxonomic lineage information was obtained for the top blast hit of each contig, and those annotated under the kingdom virus were initially identified as potential virus-associated sequences. To exclude false positives, these potential viral contigs were subjected to blastn comparisons against non-redundant nucleotide databases to distinguish viral sequences from non-viral host sequences, endogenous viral elements, and artificial vector sequences. Reference genomes of target species were selected based on blast results. Clean data aligned the reference genome of the target species using bwa (0.7.17-r1188). There were more than 50 bases aligning the reference genome defined as reads of the target species. An initial scaffold extension and reassembly from those reads were built using the *de novo* assembler SPAdes (v3.15.3). Reads aligned to the reference genome were returned to the assembled scaffold using bwa (0.7.17-r1188), and the scaffold was calibrated according to the sequencing depth of the aligned reads to output a complete sequence.

### 2.5. Phylogenetic analysis

Complete sequences obtained in this study were compared with all sequences in GenBank through blastn of NCBI, and appropriate reference sequences were selected for Phylogenetic analysis. Subsequently, MEGA_11.0.13 was used to align the sequences and reference sequences using the MUSCLE package and default parameters. The maximum-likelihood method of MEGA_11.0.13 was used to process the phylogenetic analyses with 1,000 bootstrap replicates.

### 2.6. Reassortment and variation analysis

Reassortment analysis of sequences for virus sequences was performed using SimPlot (v3.5.1). The schematic diagram of the genome structure was drawn on the basis of blastx comparison information of RNA sequence. Variation analysis was conducted through bwa, varscan, and snpeff bioinformatics software.

## 3. Results

### 3.1. Tick samples

A total of 19 live ticks were collected in Wolong Nature Reserve of Sichuan province from April 2020 to August 2020, including 17 parasitic ticks that fed on giant pandas or goats and 2 host-seeking ticks (Figure 1). These ticks were comprised of 19 adult ticks and 3 pools of tick eggs, including 4 *Ixodes acutitarsus*, 7 *Haemaphysalis longicornis*, and 8 *Ixodes ovatus*, of which 3 *Ixodes ovatus* ticks had laid eggs. Then 15 pools of tick samples, with 7 *Ixodes ovatus* from giant pandas (4 adult ticks and 3 groups of eggs), 2 *Ixodes acutitarsus* parasitizing on goats and 2 free *Ixodes acutitarsus*, and 4 *Haemaphysalis longicornis* from goats (Table 1) had total RNA extracted, followed by library preparation and sequencing for analysis.

**Table 1:** Characteristics of tick samples collected from Sichuan Province in this study.

Pool ID	Tick species	Hosts	Developmental phase	Number of ticks/eggs
PC-5	*Ixodes acutitarsus*	Goat	♀ adult	1
PC-6	*Ixodes ovatus*	Giant panda	♀ adult	1
PC-7	*Ixodes ovatus*	Giant panda	Eggs	>200 From PC-6
PC-8	*Ixodes ovatus*	Giant panda	♀ adult	1
PC-9	*Ixodes ovatus*	Giant panda	Eggs	>200 From PC-7
PC-10	*Ixodes ovatus*	Giant panda	♀ adult	1
PC-11	*Ixodes ovatus*	Giant panda	Eggs	>200 From PC-8
PC-12	*Haemaphysalis longicornis*	Goat	♀ adult	1
PC-13	*Haemaphysalis longicornis*	Goat	♀ adult	1
PC-14	*Ixodes acutitarsus*	Free	♀ adult	1
PC-15	*Ixodes acutitarsus*	Free	♀ adult	1
PC-16	*Ixodes ovatus*	Giant panda	♀ adult	5
PC-17	*Haemaphysalis longicornis*	Goat	♀ adult	2
PC-18	*Haemaphysalis longicornis*	Goat	♀ adult	3
PC-19	*Ixodes acutitarsus*	Goat	♂ adult	1

### 3.2. Meta-transcriptomes analysis

Total RNA of 15 sample pools have been analyzed by meta-transcriptomes. A total of 165.15 GB nucleotide data was generated, which were assembled and annotated for identification and characteristic of virus groups. After quality control, removal of host gene and exclusion of reads with no significant similarity to any sequence in the NCBI, 110,846 valid reads for viruses were obtained.

### 3.3. Characteristics of viruses in the tick samples

**Figure 2:**
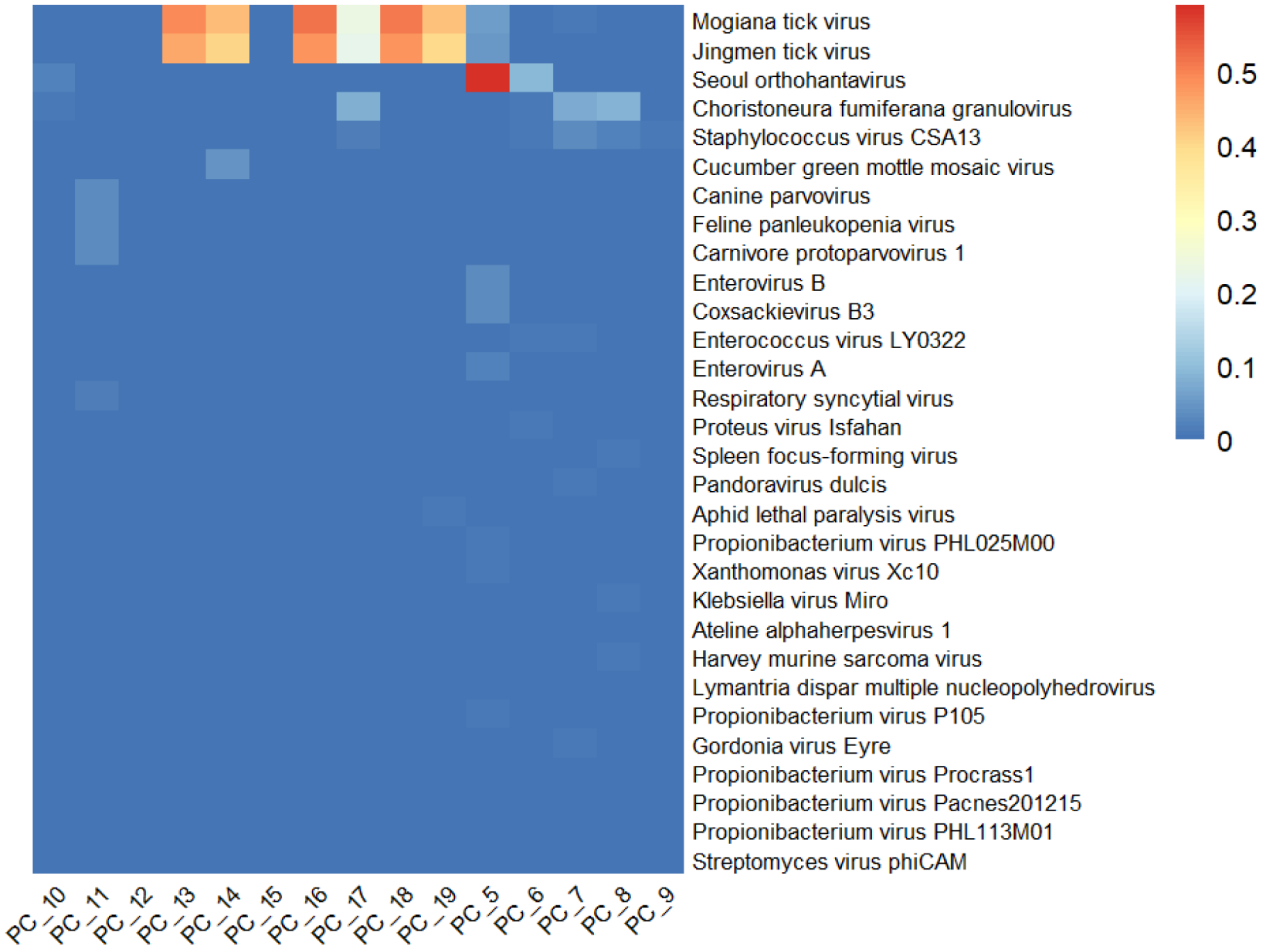
Overview of relative abundance of the top 30 virus species contained in the 15 tick sample pools from Wolong Nature Reserve of Sichuan Province.

The relative abundance of the first 30 viruses was shown in Figure 2, which represents the proportion of main viruses in every sample. According to relative abundance, the first five viruses of the tick samples were Mogiana tick virus, Jingmen tick virus, *Seoul orthohantavirus*, Choristoneura fumiferana granulovirus, and staphylococcus virus CSA13 virus from *Flaviviridae*, *Flaviviridae*, *Hantaviridae*, *Baculoviridae*, and *Rountreeviridae*, respectively. The genome of Mogiana tick virus and JMTV mainly existed in 4 tick samples, with 2 *Haemaphysalis longicornis* from goats (PC-13 and PC-18), 1 *Ixodes ovatus* from giant panda (PC-16) and 1 *Ixodes acutitarsus* from goats (PC-19), the relative abundance of which were more than 95%. Three other viruses were primarily distributed in 3 samples (PC-10, PC-5, and PC-6), 3 samples (PC-17, PC-7, and PC-8) and 3 sample (PC-17, PC-7, PC-8) separately.

Through comparison with all nucleotide sequences in GenBank database, it was found that the assembled RNA sequences in the 4 tick samples (PC-13, PC-16, PC-18, and PC-19) shared a higher similarity with JMTV, including four segments, which have been temporarily referred to as Sichuan tick virus. More significantly, the complete nucleotide sequences of the Sichuan tick viruses were obtained from the four tick samples (Table 2). These sequences have been deposited in the GenBank database with accession numbers OQ158902- OQ158905, OQ320755- OQ320766.

**Table 2:** Characteristics of complete nucleotide sequences from the following 4 tick samples.

Pool ID	Segment	Sequence length (nt)	GenBank accession No
PC-13	1	3,063	OQ320755
PC-13	2	2,796	OQ320756
PC-13	3	2,744	OQ320757
PC-13	4	2,737	OQ320758
PC-16	1	3,050	OQ320759
PC-16	2	2,672	OQ320760
PC-16	3	2,716	OQ320761
PC-16	4	2,736	OQ320762
PC-18	1	3,082	OQ158902
PC-18	2	2,679	OQ158903
PC-18	3	2,774	OQ158904
PC-18	4	2,750	OQ158905
PC-19	1	3,239	OQ320763
PC-19	2	2,753	OQ320764
PC-19	3	2,784	OQ320765
PC-19	4	2,759	OQ320766

### 3.4. Phylogenetic analysis

**Figure 3:**
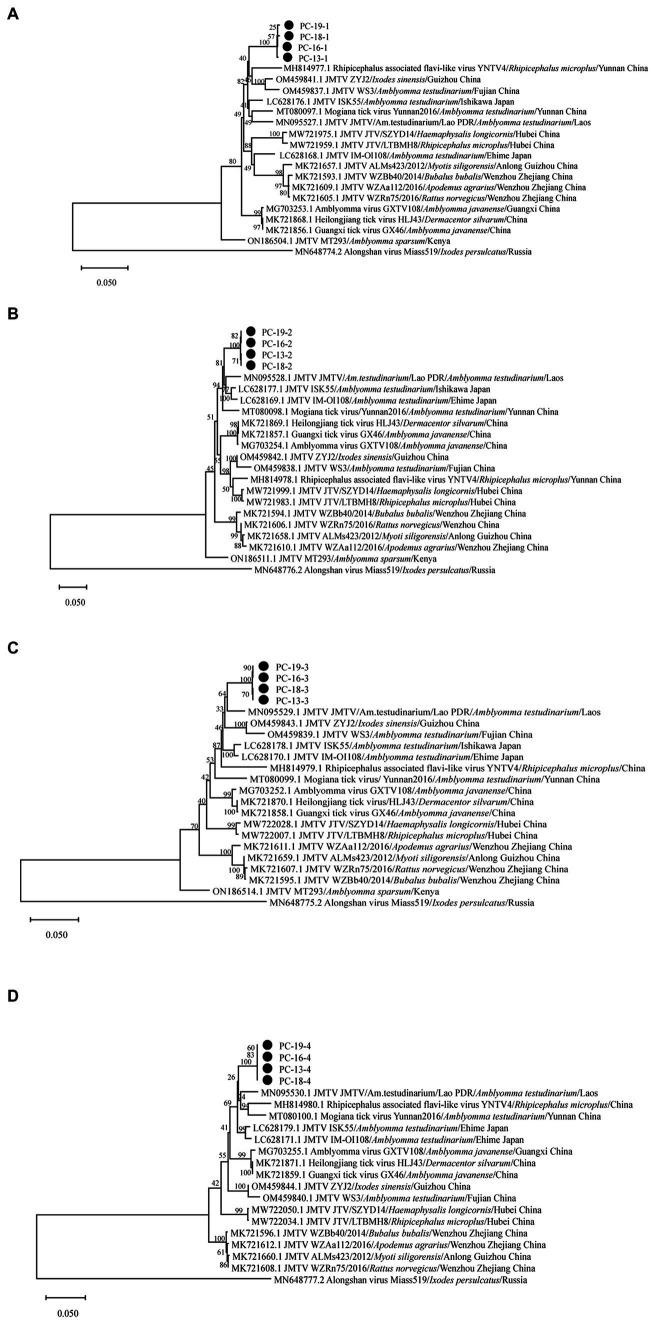
Phylogenetic analysis of the complete sequence of Sichuan tick virus in this study. Phylogenetic trees were constructed by the maximum-likelihood method using MEGA_11.0.13 with 1,000 bootstrap replicates: **(A)** (Segment 1), **(B)** (Segment 2), **(C)** (Segment 3), and **(D)** (Segment 4). The number on each branch shows the percent occurrence in 1000 bootstrap replicates.•stood for samples of Sichuan tick virus found in this study.

The sequences of Sichuan tick virus from the four samples were extremely close to each other (99.82%–100% nucleotide identity) and had high similarity (93.96%–95.71%) with *Rhipicephalus* associated flavi-like virus and JMTV strains from Lao PDR. These sequences were also found to be similar to JMTV found in Lao PDR, Brazil, Kenya and some provinces of China, with an 88.73–96.28% identity. Subsequently, the analyzed Sichuan tick viruses had an 88.77–95% nucleotide identity with Heilongjiang tick virus, Guangxi tick virus, Mogiana tick virus, Amblyomma virus and Rhipicephalus-fassociated flavi-like virus deposited in GenBank. Through the maximum-likelihood method based on four segments of Sichuan tick viruses, the phylogenetic analyses revealed that every segment of the Sichuan tick viruses clustered together, and then formed a separate clade (Figures 3A–D). Segment 1 of the Sichuan tick viruses from the four tick samples was closest to *Rhipicephalus* associated flavi-like virus strain YNTV4 from Yunnan in terms of phylogeny, with 94.64–94.66% identity (Figure 3A). Yet segments 2, 3 and 4 of Sichuan tick viruses exhibited the closest evolutionary relationship with JMTV strain from Lao PDR, with 93.96%–94.26%, 95.71%, and 95.55%–95.56% similarity, respectively (Figures 3B–D). The structure of JMTV genome was relatively conserved among all JMTV strains in the GenBank database. Segment 1 and segment 3 of JMTV encoded NSP1 and NSP2 proteins respectively, non-structure proteins, which seem to have homology with NS5 and NS3 proteins of the genus flavivirus. Segment 2 and segment 4 of JMTV encoded VP1, VP2, and VP3 proteins, structure proteins, which have no known homologs.

### 3.5. Reassortment analysis

**Figure 4:**
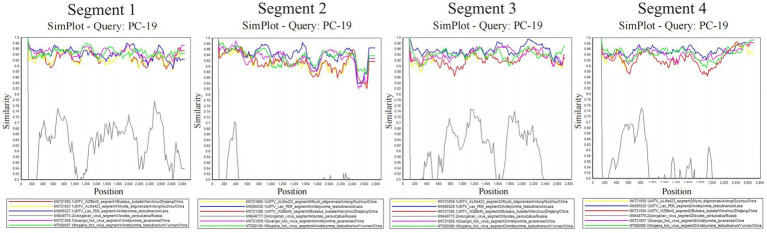
Recombination between Sichuan tick virus (PC-19) and reference sequences.

The signals of reassortment were observed between the four segments of the Sichuan tick viruses and other JMTV strains from *Amblyomma testudinarium* (Japan and Laos), *Bubalus bubalis* (China), *Myotis siligorensis* (China), *Haemaphysalis longicornis* (China). Especially, the signals of recombination between the four segments of the Sichuan tick viruses and GXTV (Guangxi tick virus) from *Amblyomma javanense* in China, MGTV (Mogiana tick virus) from *Amblyomma testudinarium* in China, suggested cross-species transmission and co-infection of segmented flavi-like viruses among multiple tick hosts (Figure 4). Reassortment has been frequently observed in segmented viruses and is considered a primary mechanism for interspecies transmission and the emergence of novel strains (Vijaykrishna et al., 2015; Ogola et al., 2022).

### 3.6. Variation analysis

**Figure 5:**
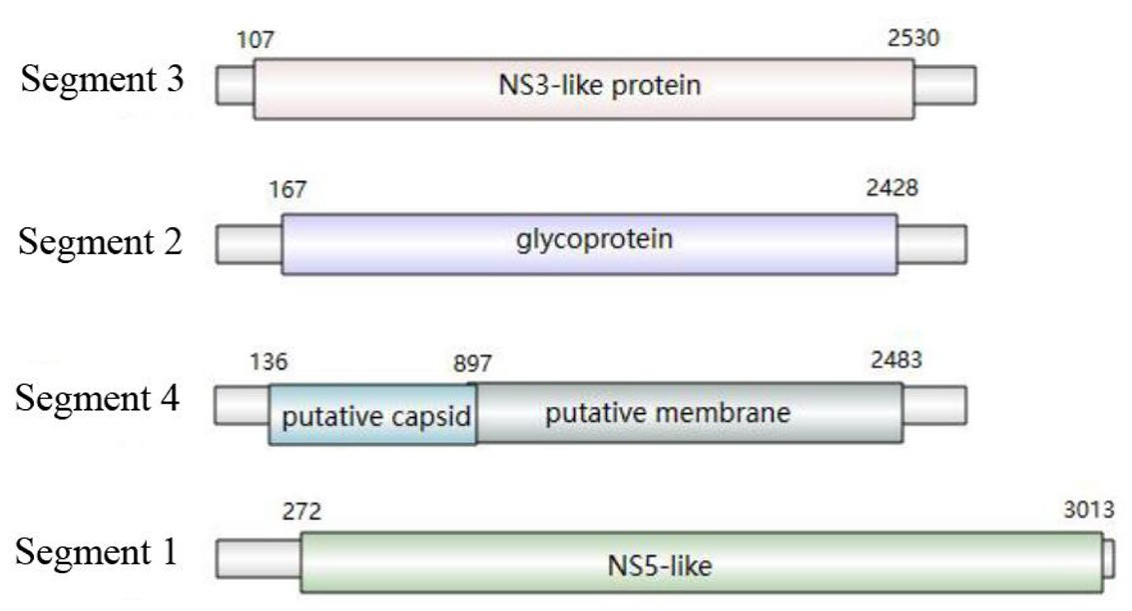
Genome structure of Sichuan tick virus (PC-19 strain).

**Figure 6:**
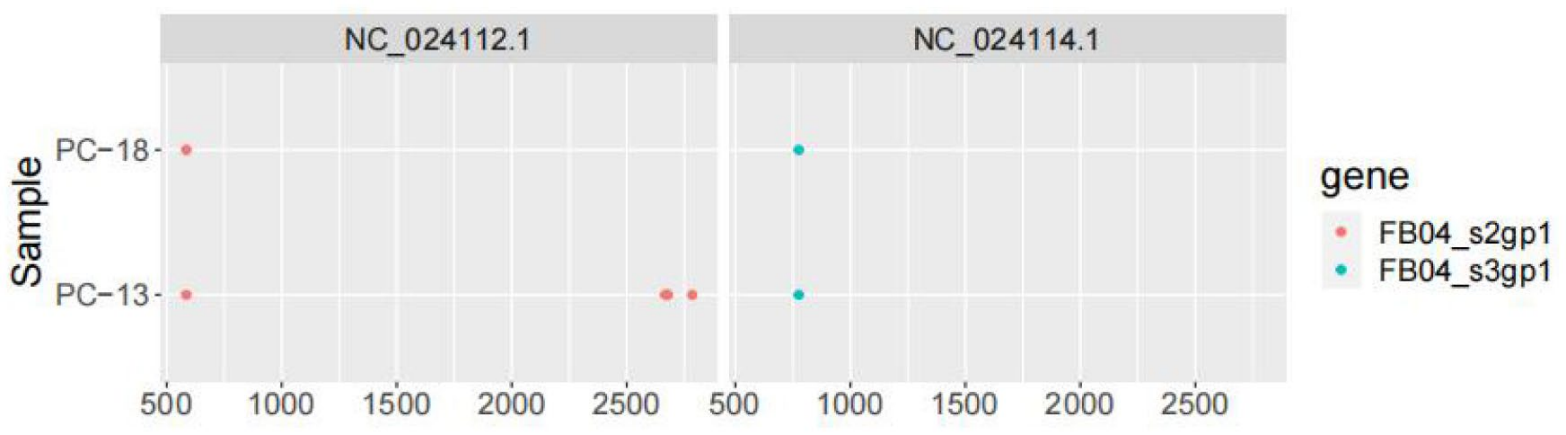
Variation of Sichuan tick virus (PC-13, PC-16, PC-18, and PC-19).

The genome structure was drawn by taking the sample PC-19 strain as an example, which can be seen in Figure 5. Segment 3 encodes NS3-like protein; segment 2 encodes glycoprotein; segment 4 consists of putative capsid protein and putative membrane protein. Segment 1 comprises NS5-like protein. After comparison and statistics by bioinformatics software, PC-19 was used as the reference standard to obtain the unique variation results among the strains (Table 3 and Figure 6). PC-13 strain has 6 unique mutations, 2 insertions and 4 point mutations. PC-18 has only 2 point mutations. The result of PC-16 variation was consistent with PC-19.

**Table 3:** Results of variation among Sichuan tick viruses.

Virus	CHROM	POS	REF	ALT
PC-13	NC_024112.1	2,668	C	CAGAAAAAAAAAAA
PC-13	NC_024112.1	2,676	G	GA
PC-13	NC_024112.1	585	A	C
PC-13	NC_024112.1	2,677	G	A
PC-13	NC_024112.1	2,785	C	A
PC-13	NC_024114.1	776	T	C
PC-18	NC_024112.1	585	A	C
PC-18	NC_024114.1	776	T	C

## 4. Discussion

Since the discovery of Jingmen tick virus (JMTV), JMTV and Jingmenviruses group have been found to be distributed across the world, while there is little research on them in Sichuan Province. In this study, we described the discovery of Sichuan tick virus (SCTV), a novel JMTV-like virus, in three tick samples collected from goats and one from a giant panda. This is the first record of a JMTV-like virus in Sichuan Province.

To our knowledge, tick species that have been shown to carry JMTV are from 26 species in 6 genera: *Rhipicephalus, Amblyomma, Dermacentor, Haemaphysalis, Hyalomma,* and *Ixodes* (Shi et al., 2016). Remarkably, the SCTV genome showing strong similarity to JMTV in this study was first found in several tick species that were not previously associated with JMTV. The special tick species contained *Ixodes ovatus* parasitizing on a giant panda and *Ixodes acutitarsus* that fed on goats, which has expanded the range of tick hosts associated with JMTV. This in turn has increased the risk of human and wild animals infected with JMTV. When combined with earlier research, these results indicate that JMTV is an emerging tick-borne virus with an extensive geographical distribution and is capable of infecting a significant variety of tick species.

Meanwhile, SCTV was identified for the first time in all 3 tick species collected in Wolong Nature Reserve of Sichuan province, suggesting that SCTV exists in various hosts, may be widely distributed throughout southwestern China and form a stable ecological cycle in the Wolong Nature Reserve. Moreover, the 4 tick samples infected with SCTV were engorged ticks collected from a giant panda and goats, suggesting that SCTV also possibly infected parasitic animals. It would appear probable that the risk of SCTV transmission between ticks and parasitic animals could be increased by the act of co-feeding in different animals, especially national protected animals. More seriously, human contact with wild animals (such as giant pandas and goats) may increase the incidence of zoonosis related to SCTV and the hazard of human and wildlife infection with SCTV.

Phylogenetic analysis regarding Sichuan tick virus showed the four segments of SCTV had the closest evolutionary relationship with *Rhipicephalus* associated flavi-like virus strain YNTV4 from Yunnan province and JMTV strain from Lao PDR, illustrating that these jingmenviruses in special geographical locations (Laos, Sichuan and Yunnan provinces) manifested a consistent evolutionary direction under similar environmental factors (similar climate and ecological environment), or that they share a common virus ancestor. JMTV appears to be highly conserved in terms of its evolution as a concerned virus. However, reassortment analysis results demonstrated the sequence differences between SCTV and *Rhipicephalus* associated flavi-like virus, JMTV, were associated with gene recombination. In addition, although SCTV, a JMTV-like virus, showed the highest similarity with JMTV strains ISK55, IM-OI96, and IM-OI108 from Japan, SCTV phylogenetically displayed the closest relationship with *Rhipicephalus* associated flavi-like virus from Yunnan and JMTV from Laos, manifesting that may be the evolutionary consequences of different JMTV lineages or extensive mutations of the genome. Furthermore, the sequences of SCTV were remotely different from other jingmenviruses, with ALSV, HLJTV, *Amblyomma* virus, and Guangxi tick virus from China and Russia, suggesting that the gene recombination between flavivirus and other segmented flavi-like viruses was very common.

This study covered the discovery of Sichuan tick virus in Wolong Nature Reserve of Sichuan Province, which can increase our understanding of tick-borne viruses in the area, provide early warning and prediction of possible tick-borne diseases, reduce misdiagnosis and delay in treatment of tick-borne diseases. It can also improve the diagnostic rate and contribute to the survival of local residents. At the same time, tick-borne viruses in the area were investigated to prevent the occurrence of viral tick-borne infectious diseases, which is beneficial to the safety of residents and is also of great public health significance. Furthermore, early discovery of tick-borne viruses, their vectors, and hosts can provide support for the prevention and control of relevant tick-borne diseases, thereby protecting humans and wildlife, which is significant for public health.

Limitations of this study are as follows. Firstly, the study lacks PCR’s validation of SCTV and its serological evidence in humans or animals. Secondly, Sichuan tick virus has not yet been isolated from ticks. Thirdly, the small number of ticks collected in this study is insufficient to indicate the prevalence of JMTV in Sichuan Province, China. In the future, a large-scale epidemiological survey of JMTV needs to be conducted in Sichuan Province to understand more relevant details, such as distribution, hosts, and prevalence. In addition, further serological testing is needed to assess Sichuan tick virus pathogenicity.

## 5. Conclusion

In summary, we discovered and confirmed one new Jingmen tick virus, Sichuan tick virus, in ticks parasitizing protected animals and livestock, in the Wolong Nature Reserve of Sichuan Province, and its complete nucleotide sequences have been obtained. However, further investigation is required to determine the pathogenicity of Sichuan tick virus to humans and animals, as well as its epidemiologi-cal characteristics in nature.

## Data availability statement

The data presented in the study are deposited in the Genbank of National Center for Biotechnology Information, accession number OQ320755-OQ320766 and OQ158902-OQ158905.

## Author contributions

JJ and XR conceived and designed the experiments. LH, BJ, and LX performed the experiments. WC, LC, SL, and LH analyzed the data. JJ, FW, PY, HT, and QZ were implemented sample collections. LH and JJ drafted and reviewed the manuscript. All authors contributed to the article and approved the submitted version.

## Funding

This work was supported by the State Key Research Development Program of China (2019YFC1200501).

## Conflict of interest

LC is employed by Beijing Macro & Micro-test Bio-Tech Co., Ltd.; WC is employed by BGI PathoGenesis Pharmaceutical Technology.

The remaining authors declare that the research was conducted in the absence of any commercial or financial relationships that could be construed as a potential conflict of interest.

## Publisher’s note

All claims expressed in this article are solely those of the authors and do not necessarily represent those of their affiliated organizations, or those of the publisher, the editors and the reviewers. Any product that may be evaluated in this article, or claim that may be made by its manufacturer, is not guaranteed or endorsed by the publisher.
