# Erratum: Identification of novel Jingmen tick virus from parasitic ticks fed on a giant panda and goats in Sichuan Province, southwestern China

**DOI:** 10.3389/fmicb.2023.1279784

**Published:** 2023-08-31

**Authors:** Frontiers Production Office

**Affiliations:** Frontiers Media SA, Lausanne, Switzerland

**Keywords:** Jingmen virus group, tick borne disease, Sichuan tick virus, Sichuan Province, giant panda

Due to a production error, an additional affiliation was erroneously listed for author Shunshuai Liu. The affiliation “Lanzhou Veterinary Research Institute, Chinese Academy of Agricultural Sciences, Lanzhou, China” has been removed from the list of author affiliations.

There was a missing affiliation for the Review Editor Guangyuan Liu. The following affiliation should be inserted “Chinese Academy of Agricultural Sciences, China.”

The publisher apologizes for these mistakes. The original article has been updated.

